# Analysis of free chlorine in aqueous solution at very low concentration with lateral flow tests

**DOI:** 10.1038/s41598-019-53687-0

**Published:** 2019-11-20

**Authors:** K. Uta Schwenke, Dieter Spiehl, Marcel Krauße, Laura Riedler, Anna Ruppenthal, Klaus Villforth, Tobias Meckel, Markus Biesalski, Daniel Rupprecht, Gerhard Schwall

**Affiliations:** 1 Merck Lab @ TU Darmstadt, Alarich-Weiss-Strasse 8, D-64287 Darmstadt, Germany; 2 Science Relations, Merck KGaA, Frankfurter Strasse 250, D-64293 Darmstadt, Germany; 3 Printing Science and Technology, Technische Universität Darmstadt, Magdalenenstrasse 2, D-64289 Darmstadt, Germany; 4 Macromolecular Chemistry and Paper Chemistry, Technische Universität Darmstadt, Alarich-Weiss-Strasse 8, D-64287 Darmstadt, Germany; 5 Paper Technology and Mechanical Process Engineering, Alexanderstrasse 8, Technische Universität Darmstadt, D-64283 Darmstadt, Germany; 6 Advanced Analytics, Merck KGaA, Frankfurter Strasse 250, D-64293 Darmstadt, Germany

**Keywords:** Environmental monitoring, Health care

## Abstract

Test strips are convenient tools for rapid, semi-quantitative analysis of a variety of parameters by dipping them for a few seconds in a sample solution followed by a simple colorimetric read-out. Their sensitivity is mainly determined by the reactivity of the test dyes on the reaction zone and is not sufficient for some applications. The detection limit of commercially available free chlorine test strips, for example, is at present not low enough to confirm the absence of this analyte as disinfectant in rinsing solutions after disinfection or to control required residual amounts of chlorine in drinking water. Therefore, we developed a user-friendly lateral flow test which is capable to detect very low amounts of free chlorine. The latter relies on a larger sample volume passing the reaction zone as compared to simple dip test strips. An amount of as low as 0.05 ppm chlorine can, however, only be detected if oxidation stable flow test substrates are used. The eventually developed flow test reaches a 10x higher sensitivity than a commercial dip test. The result is obtained within 4–5 min flow time, whereby no action is required by the user during this analysis time.

## Introduction

Test strips are mobile laboratories, which allow for semiquantitative analysis of ions, organic and inorganic substances anywhere by just dipping them for a few seconds in the sample solution. In their simplest form, these devices consist of a plastic handle with a reaction zone composed of a few square millimeters of paper impregnated with specific test reagents. In contact with an analyte containing solution, a colour change of the test reagent occurs, whereby the intensity of the colour can be compared to a colour card to estimate the analyte concentration. The colour intensity can further vary with contact time of the test in the solution, which requires precise timing for quantitative results. The sampled volume is intrinsically defined by the pore volume of the paper used as reaction zone. This makes test strips very robust analytical systems, but the small sample volume leaves little room to adjust their detection range, which is hence mainly determined by the characteristics of the test reagents.

**Figure 1:**
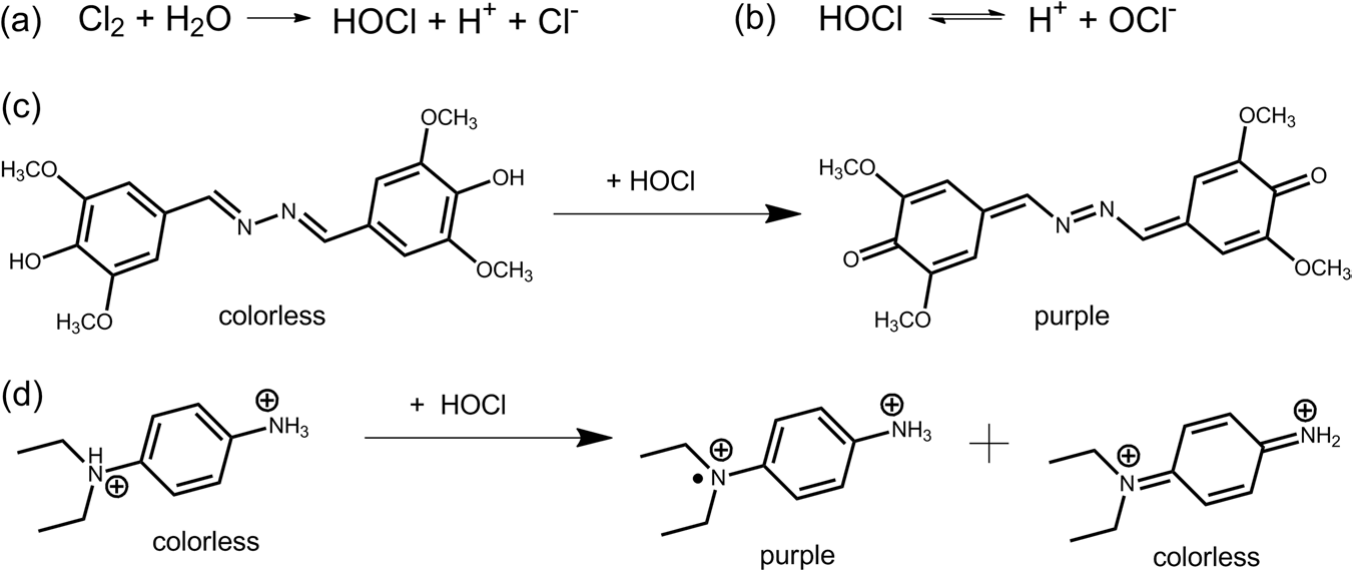
(**a**) Chlorine gas hydrolyses in water to hypochlorous acid. (**b**) The ratio of hypochlorous acid to hypochlorite ion varies in dependence of the pH in aqueous solution. Both species are referred to as “free chlorine”. (**c**) Oxidation reaction of syringaldazine (3,5-dimethyl-4-hydroxybenzaldazine) and (**d**) N,N-diethyl-p-phenylenediamine (DPD) with free chlorine.

Due to limited sensitivity of the used reagents, certain analyses, which require the detection of very low analyte amounts, are not conducted with dip test strips but with more complicated methods which require handling of liquids like photometry. Test strips for redox parameters such as free chlorine (chlorine Cl_2_, hypochlorous acid HOCl or hypochlorite ion ClO^−^ depending on pH value^1^), peracetic acid and hydrogen peroxide have typically a detection limit between 0.5 and 5 ppm^2,3^. This sensitivity is not sufficient to analyse for example rinsing solutions after the disinfection of food and beverage production facilities to be disinfectant free. The disinfection of drinking water with chlorine requires also the determination of very low residual free chlorine amounts: The World Health Organization recommends a residual amount of 0.2 to 0.5 ppm at the point of delivery^4^. According to the German Ordinance on Potable Water^5^, the free chlorine content in drinking water should be even adjusted between 0.1 and 0.3 ppm after the disinfection process. The most common chromophores for photometric and visual detection of free chlorine are N,N-diethyl-p-phenylene-diamine (DPD)^6^ and syringaldazine (SA)^7,8^ (reaction schemes Fig. 1). In order to increase the sensitivity of test strips, the above described volume limitation of the test zone has to be overcome. A team from Miles Laboratories suggested an absorbent wick to increase the analyte volume instead of a simple SA test patch. However, only a detection limit of 0.5 ppm free chlorine could be reached^9^. A free chlorine test containing leuco crystal violet (LCV) adsorbed on silica gel, which was described in the same patent, reached a detection limit of 0.2 ppm^9^. LCV reacts, however, slowly with bound chlorine and is hence less specific than SA^10^. Another typical redox dye for testing disinfection parameters is tetramethylbenzidine (TMB). In order to overcome the detection limit of dip test strips, Ramana *et al*. suggested to increase the contact time of the dye with the analyte by prolonged stirring of the test strip in the analyte solution^11,12^. A detection limit of 0.05 ppm was reached after stirring for 90 s using TMB, and of 0.2 ppm with a mixture of SA and vanillinazine after 60 s. A drawback of the prolonged stirring method is the need for precise timing and reproducible stirring as well as bleeding of reagents in the sample solution.

The aim of our work presented here was to address the above outlined challenges with respect to higher sensitivities in free chlorine dip test strips. We therefore designed and investigated a paper-based analytical lateral flow test, which transports the sample through the dye containing reaction zone. As the latter is positioned outside the sample solution, the dye does not bleed into the analyte solution. Moreover, an increased and defined volume passing the reagent (i.e. sensor) zone on the test-strip leads to a higher sensitivity in comparison to a conventional dip test without additional handling constraints for the user besides a somewhat longer waiting time. The pore volume and length of the paper-based flow substrate defines thereby the analyte volume and the test time. One drawback using the flow principle consists, however, in the reactivity of many substrate materials, including cellulose and hence paper, with the strong oxidizing agents^13^. Especially low amounts of analyte are consumed during the flow to the reaction zone leading to false negative results. Therefore, several flow substrates were screened for reactivity towards chlorine in order to reach very low detection limits. Unfortunately, it seems that substrates which are more stable against oxidation, do not retain the dye sufficiently on the substrate during the flow of the analyte. Two different approaches were developed to circumvent this limitation: First, a hybrid flow test consisting of an oxidation stable material which dips in the analyte solution and an attached paper substrate with high retention of the dye. Second, a flow test consisting of an oxidization stable substrate on which the dye is immobilized with cellulose fines or cellulose acetate, respectively.

## Methods

### Substrate materials

Fibrous cellulose, polymer and glass fibre materials were investigated as flow test substrates: lab-made cotton linters paper, cotton linters paper 2992 by Schleicher & Schüll (180 g/m²), dried blood spot paper (DBS) TFN by Ahlstrom-Munksjö, polyester fibre material (PET 6613, 6613 H and 6614) by Ahlstrom-Munksjö (100, 100 and 75 g/m²), Spec-Wipe 3 with 45% polyester and 55% cellulose by VWR (67 g/m²), glass fibre (GF) 691 by VWR (52 g/m²) and glass fibre MN 85/90 BF by Macherey-Nagel (90 g/m²).

All lab-made paper sheets of unmodified and pre-oxidized (see below) cotton linters fibres were made with a grammage of approximately 180 g/m² using a Rapid-Köthen sheet former (Estanit) according to DIN 54358 and ISO 5269/2. No additives or fillers were used for paper making.

The cotton linters pulp (Grade 225 HSR-M, Buckeye Technologies) was pre-oxidized prior to paper making with free chlorine and with a TEMPO-catalyzed oxidation. Therefore, 20 g cotton linters fibres were disintegrated in approximately 1.5 l water. For the oxidation with free chlorine the fibre suspension was mixed after disintegration with 12.39 g dichloroisocyanuric acid sodium salt dihydrate (GR for analysis, Merck KGaA, 4.84 mmol_HOCl_/g_pulp_, approx. 4500 ppm chlorine). The suspension was adjusted with HCl to pH 5 and stirred for 4 h. For the TEMPO-catalyzed oxidation, the fibre suspension was mixed with 500 mg sodium bromide (NaBr, Roth, 99%), 50 mg (2,2,6,6-Tetramethylpiperidin-1-yl)oxyl (TEMPO, Roth, 98%) and 49.2 ml sodium hypochlorite solution (NaOCl, 12%, Roth, 4.84 mmol_NaOCl_/g_pulp_) as described by Saito *et al*.^14^ HCl was added several times during the 4 h reaction to the suspension to keep the pH between 10 and 11. Both reactions were quenched with 20 ml ethanol, before the fibres were washed twice with water, once with ethanol and again several times with water.

The paper Schleicher & Schüll 2992 was also used after hornification. For the latter, the paper was placed overnight in an oven at 120 °C with 100% ventilation. Subsequently, the paper was equilibrated under norm climate conditions according to DIN EN 20187 (ISO 187) in order to restore the water content of air-dry paper.

### Preparation of flow tests

**Figure 2:**
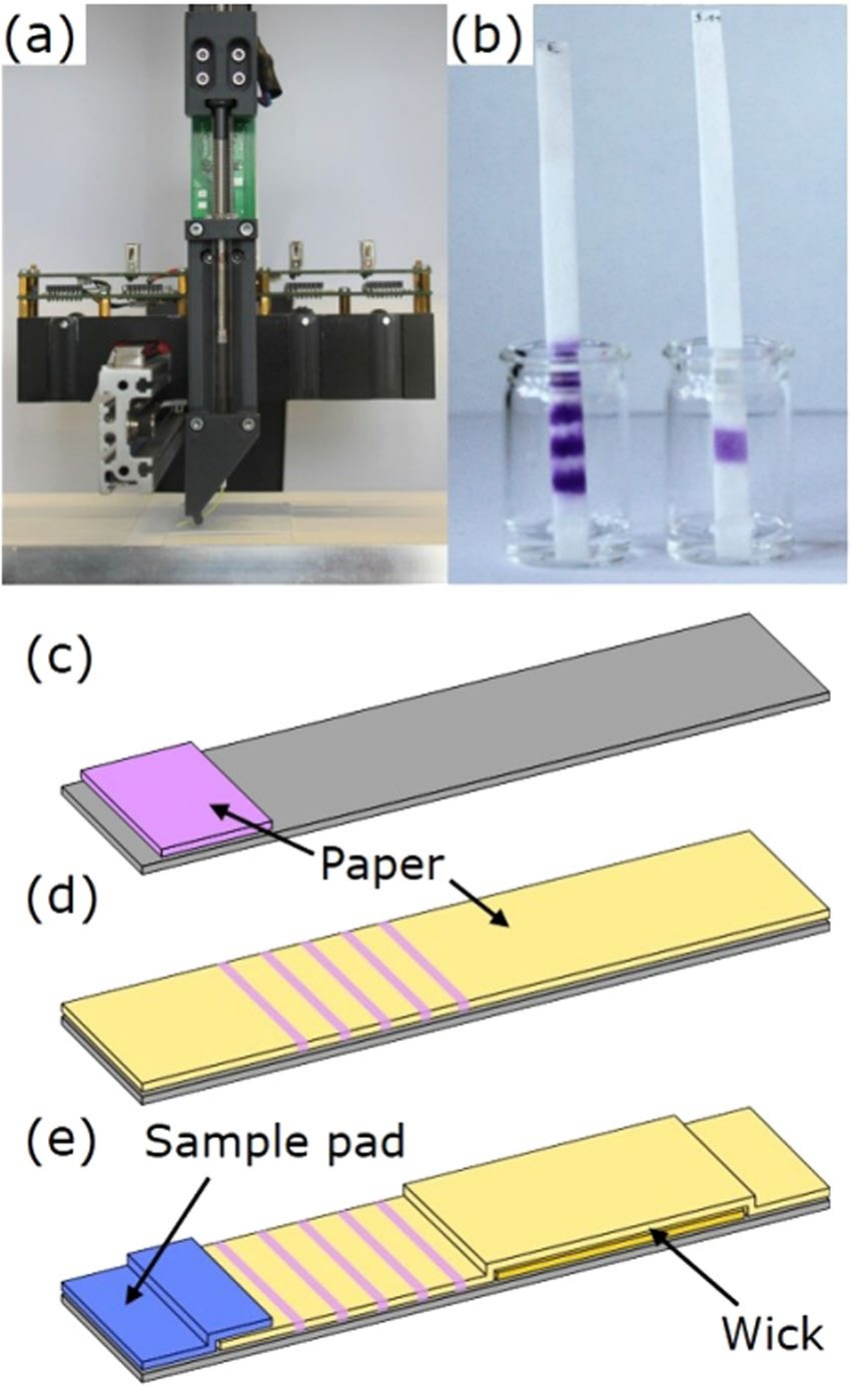
(**a**) Hyrel System30M 3D-Printer using a custom-made syringe-based print head for dispensing of the ink on the substrates. (**b**) Barcode flow test and single patch flow test while free chlorine solution flows through the tests. (**c**) Scheme of a conventional colorimetric dip test. (**d**) Scheme of a barcode flow test. (**e**) Scheme of a hybrid flow test with an inert sample pad. Please note that the schemes are not drawn to scale.

All substrates were cut to 9 cm × 5 cm pieces. Some of the substrates were immersed for 5–15 s in a phosphate buffer pH 5. The buffer was prepared by dissolving 10 g di-sodium hydrogen phosphate (Na_2_HPO_4_, >99%, Roth) in 90 g Milli-Q water and then pouring this in a solution of 40 g potassium dihydrogen phosphate (KH_2_PO_4_, 98%, Roth) in 160 g water until reaching pH 5. The substrates were dried overnight on a glass plate at room temperature. All substrates were glued on backing cards supplied by Lohmann Precision Die Cutting (LC-58717, polystyrene with GL-187 acrylic PSA) for better stability of the tests when wet. Between some backing cards and substrates, an additional wick (GF MN 85/90 BF, PET 6613 H, cotton linters paper 2992 or extra thick blot paper by Bio-Rad Laboratories) of 4.5 cm × 5 cm was glued leaving 1 cm free from the top edge. For hybrid tests with a chlorine stable material for sample contact (sample pad) with a size of 1.2 cm × 5 cm, wicking pads with only 4 cm length were used and paper substrates with a size of 8 cm × 5 cm in order to obtain an overlap of 1–2 mm with the sample pad (cf. Fig. 2e).

Printing was conducted on a Hyrel System30M 3D-Printer with a custom-made syringe-based print head (printing setup see Fig. 2a), which was used with 250 µl or 500 µl syringes (Type 1725 or 1750 TLLX, Hamilton). Standard inks consisted of 2 mM syringaldazine (SA, 99%, Sigma-Aldrich) solution in absolute ethanol. Simple flow tests were prepared by printing this ink with a bent stainless-steel needle of 1.19 mm diameter with a dosing of 4 × 2 µl/cm at 1.5 cm from the bottom edge of the test. Barcode flow tests (scheme see Fig. 2d) were prepared by printing this ink with a flexible polypropylene needle (length 1″, inner diameter 0.25 mm, needle hold at 20°) with a dosing of 4 × 0.5 µl/cm at 1; 1.5; 2; 2.5 and 3 cm from the bottom edge. For hybrid tests (scheme see Fig. 2e), the SA ink was printed at 1.4; 1.9; 2.4; 2.9 and 3.4 cm from the bottom edge due to the size of the sample pad.

In order to increase the retention of SA on PET and GF substrates, the ink was modified with cellulose fines and polymers.

Cellulose fines Arbocel BE600-10-TG (short fibres, 18 µm × 15 µm, 99.5%) and Arbocel UFC100 (ultrafine powder, 6–12 µm, 99.5%) by J. Rettenmaier & Söhne GmbH + Co KG were added with 1, 2 or 4% (m/v) to an already dissolved ethanolic 2 mM SA solution (e.g., 15 mg in 1.5 ml). All inks with cellulose fines were dispersed with an ultrasound homogenizer (UW2070, Bandelin) for 2 × 1 min and then printed with a flexible polypropylene (PP) needle (length 1″, inner diameter 0.6 mm, needle hold at 20°) with the dosing of 1 × or 2 × 2.5 µl/cm at 1; 1.5; 2; 2.5 and 3 cm from the bottom edge of the PET 6613 H substrate.

Cellulose acetate (CA, CA-398–30, Eastman) was added with 0.3 to 5% (m/v) to an already dissolved 2 to 10 mM SA solution in acetone (99.9%, VWR). All inks were printed with a flexible PP needle (length 1″, inner diameter 0.25 mm, needle hold at 20°) with the dosing of 4 × 0.5 or 1 × 2 µl/cm at 1; 1.5; 2; 2.5 and 3 cm from the bottom edge of the PET 6613 H or GF MN 85/90 BF substrate.

After printing, all substrates were cut to 0.5 cm wide individual tests. For comparison, conventional colorimetric chlorine dip test strips (1.17925, MQuant, Merck KGaA) were used as received (scheme Fig. 2c).

### Analysis of flow tests

A 1000 ppm stock solution of free chlorine was prepared by dissolving 92.5 mg dichloroisocyanuric acid sodium salt dihydrate (GR for analysis, Merck KGaA) in 50 ml Milli-Q water on the day of analysis. The stock solution was diluted to 5 and 2 ppm. Solutions with lower concentrations were prepared by diluting the 5 ppm solution. All used glass vials and flasks were pre-rinsed with the respective solution. The flow tests were run in 0.5 ml of the respective solution in a 3 ml vial (Fig. 2b), whereby the level of the sample solution is below the SA zones on the test. After removal of the flow tests from the solution, they were immediately scanned with the scanner CanoScan 9000 F MarkII (Canon, 600 dpi without any image correction). The intensity of the purple colour of the oxidized SA was determined by ImageJ 1.94 v using the colour deconvolution plugin^15^. The RGB stain vector was determined to [0.38 0.89 0.24] on paper and [0.37 0.86 0.35] for PET and GF and the RGB intensity value determined from the deconvoluted “purple” image, on which only this colour remained in order to avoid interference from grey background due to water stains on the substrate.

### Analysis of chlorine consumption

In order to determine the amount of chlorine consumed by the different substrates, 8 ml of 2 ppm chlorine solution (cf. previous section) was added to 96 mg of cut substrate pieces of approx. 0.3 cm². After a certain amount of time between 1 min and 180 min, the supernatant was filtered with a 0.2 µm PP syringe filter (VWR) and stained using the liquid reagents from the Merck test kit for free and total chlorine determination (1.14801, Merck KGaA). 6 ml of the filtrate was added to 3 drops of solution 1 (DPD dye) and 1 drop of solution 2 (sulphuric acid) of the chlorine test kit. Sample spectra were collected in PMMA macro cuvettes with a Cary 60 UV/Vis spectrometer (Agilent) in a wavelength range from 400–600 nm with 600 nm/min scan rate. Milli-Q water was used to determine the baseline. The chlorine concentration was determined by the Beer-Lambert law using the absorbance at 551 nm (ε = 21000 l mol^−1^ cm^−1^)^16^. To determine the time stability of the chlorine solution, the chlorine concentration of the initial 2 ppm solution was determined before and after all measurements of the day using the test kit for free and total chlorine determination.

## Results

### Performance of Cl_2_ flow tests in comparison to a conventional dip test

First, we compared a commercially available free chlorine dip test from Merck with a simple paper-based flow test to investigate the general influence of a capillary driven flow on the sensitivity of the test, i.e. to address the question, whether a prolonged exposure of the dye to the analyte solution leads to enhanced sensitivity.

**Figure 3:**
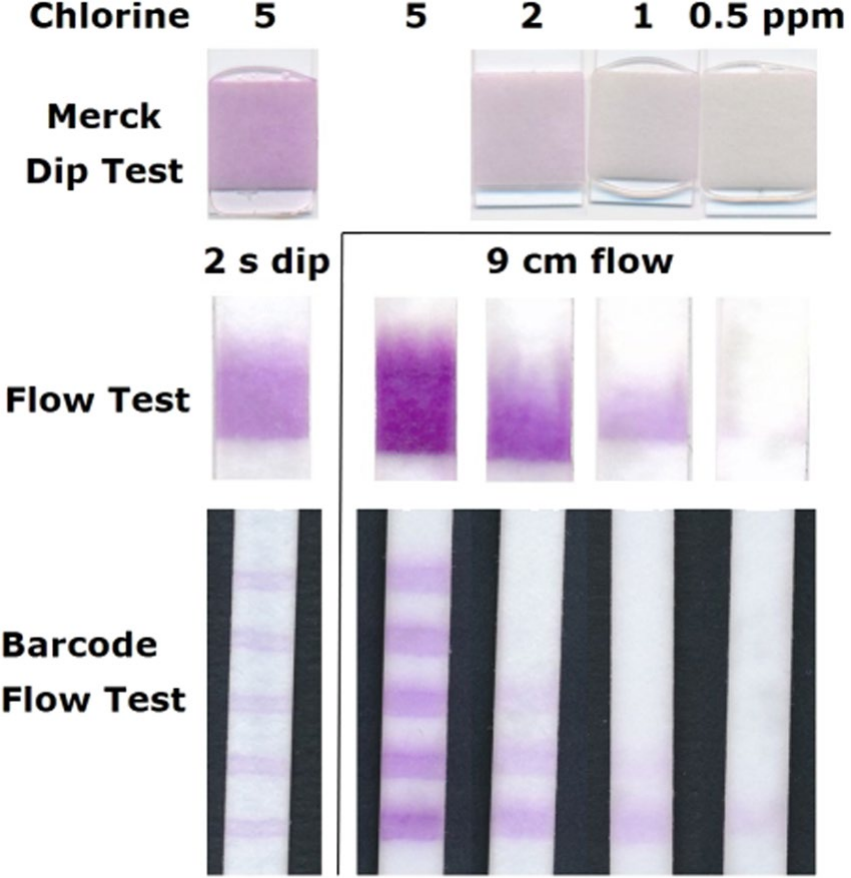
Images of commercial free chlorine test strips after 2 s dip in free chlorine solution of 5 to 0.5 ppm in comparison to 9 cm long flow tests and barcode flow tests after 2 s dip in 5 ppm solution or after solutions of 0.5 to 5 ppm free chlorine flowed through the test for approximately 7:30 min or 5:30 min respectively. Please note that the shorter flow time for the barcode flow test in comparison to the simple flow test arises due to a change of the paper orientation towards the faster machine direction.

For that, a broad pad of SA ink was printed on a cotton linters paper of 9 cm length without any further additives. Figure 3 shows that the dip of the reaction zone of this paper strip in a 5 ppm chlorine solution leads to a colour intensity comparable to the commercial dip test. Placing the flow test, however, only in a small amount of analyte solution which does not cover the reaction zone immediately but flows by capillary forces within 7–8 min to the end of the 9 cm long paper strip, leads to a clearly higher colour intensity compared to the commercially available dip test. Next to the colour intensity, the width of the coloured pad also depends on the chlorine concentration. For a better readability, it is helpful to use a sheath around the paper strip with an aperture, as was suggested by Bauer *et al*.^9^, that the user focuses on the colour intensity to interpret the results. Alternatively, it is possible to print an even broader dye pad and use a distance-based detection readout^17–19^. However, the coloured section is not homogeneous and fades towards the end, making its length hard to measure. Printing separate dye lines in a barcode flow test^20–23^ instead of one dye section, facilitates the readout by eye as both the colour intensity and the number of lines can be compared for semiquantitative analysis. Figure 3 (bottom) shows scans of such barcode flow tests with five lines after contact with different chlorine solutions. Again, the colour of the flow test is much more intense than of the dipped test. Finally, resolution with respect to different concentrations is enhanced by the barcode setup in comparison to a simple flow test. Hence, these results suggest that a paper-based flow test leads to a higher sensitivity than a dip test, and, additionally, a flow test allows for more complex printing patterns which may simplify a semi-quantitative analysis.

**Figure 4:**
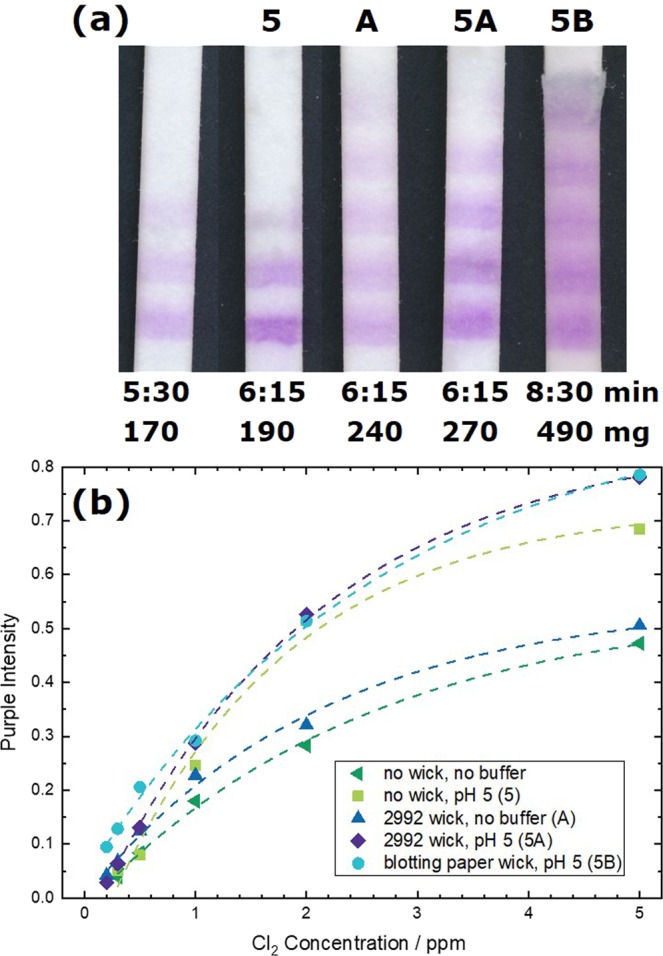
(**a**) Images of barcode flow tests after 2 ppm free chlorine solution flowed through the tests for the time below the images. The mass written below the images relates to the amount of sample absorbed by the test, which was determined by weighing. The substrate for tests marked with “5” was impregnated with buffer pH 5 prior printing. Tests marked with “A” had an additional 4.5 cm long wick made of 2992 Schleicher & Schüll paper (430 µm thickness, 180 g/m²), tests marked with “B” had a wick made of Biorad blotting paper (2.45 mm thickness, 734 g/m²). (**b**) Intensity of the purple SA dye in dependence of the chlorine concentration extracted from the first line of tests such as shown exemplary for 2 ppm in (**a**). The dashed lines were integrated for guidance of the eye and were obtained by an asymptotic exponential fit (y = a − bc^x^) of the data sets.

While 0.5 ppm chlorine can only be barely detected with the commercial dip test, this concentration is clearly visible for the hereby proposed flow tests. So far, the ink consisting of the dye in ethanol was simply printed on a cotton linters paper without any further additives. The pH is for example especially important for the oxidation of SA. It was found that the pH is preferably set to 6.0 to increase both colour response and sensitivity^7,8^. Hence, we impregnated the paper with a phosphate buffer solution of pH 5 or 6. Indeed, a clear intensity increase was observed for tests treated with buffer. However, the increase was the same for pH 5 and 6. Since impregnation with buffer pH 6 doubled the flow time, whereby the time delay due to buffer pH 5 was less than a minute, we decided to use pH 5 buffer impregnation for further tests to yield test results as fast and sensitive as possible (cf. Fig. 4, test marked “5”). The sensitivity of flow tests can be further increased by processing higher volumes^24^, which can for example be achieved by longer test strips^25^, a wider sample pad^26^ or a fan-shaped wicking pad^27^. An even simpler way to increase the amount of sample passing the reaction zone, consists in the addition of a wick after the reaction zone glued in between the test strip and the backing card (cf. Fig. 2e). When the same material is used for the wick and the test strip, the sample volume capacity is doubled for the length of the wick. As we use wicks half the length of the flow test, 1.5 times of analyte volume is sampled. Figure 4a displays tests after analysis of 2 ppm chlorine solution. More barcode lines are coloured for tests with additional wicks than without (Fig. 4a, test marked “A”). Combining buffer and wicking pads leads to a higher number of coloured lines with more intense colour (Fig. 4a, test marked “5A”). Using an extra thick blot paper as a wick (Fig. 4a, test marked “5B”) increases the amount of sample analysed further (sample masses mentioned in Fig. 4a). However, too high of a sample flow leads to bleeding of the dye and less readable results. Furthermore, the flow time was substantially longer in this case due to the slower flow in the blot paper.

In order to compare the effect of buffer and additional wick over the detectable range of free chlorine, the intensity of the purple colour of the first line was extracted and plotted in Fig. 4b. Remarkably, the colour intensity increase due to buffer impregnation is very strong for high chlorine concentration, but negligible for the more interesting low end. The effect of the colour intensity increase related to the wick is relatively constant over the full range, although Miller *et al*. found that the sensitivity increase due to increased sample volume is less for low concentrations^24^. The improvement due to the wick in our analysis seems rather small. However, it is important for the low end as the main improvement of the wick is the increased number of coloured stripes and not the colour intensity which is plotted in Fig. 4b. In conclusion, it is at best possible to detect down to 0.2 ppm free chlorine using flow tests with an additional wick. Please note that 0.2 ppm free chlorine caused a visually observable purple hue on the test, but it is not possible to determine a rigid limit of detection on that visual inspection. To do so, a larger field study involving multiple untrained testers would be necessary which is out of scope of this proof-of-principle study.

### Cl_2_ consumption by flow test substrates

As discussed above, a flow test set-up substantially increases the sensitivity of the chlorine analysis. However, there is also a drawback of the prolonged contact between analyte and test: most strong oxidizing agents such as free chlorine do not only oxidize the redox dye but also interact with most of the potential substrate materials, including cellulose as the major molecular component of the used paper sheets^13^. Trace amounts of chlorine may be consumed by reaction with the substrate material while flowing to the reaction zone, which may limit the sensitivity to 0.2 ppm free chlorine, as well as increase the error of the determined absolute concentration of chlorine. The latter becomes a problem, if very small free chlorine concentrations are to be detected. In order to increase the sensitivity as much as possible, a more oxidation stable flow substrate material has to be selected, and stability of the substrate material against oxidation processes needs to be evaluated.

**Figure 5:**
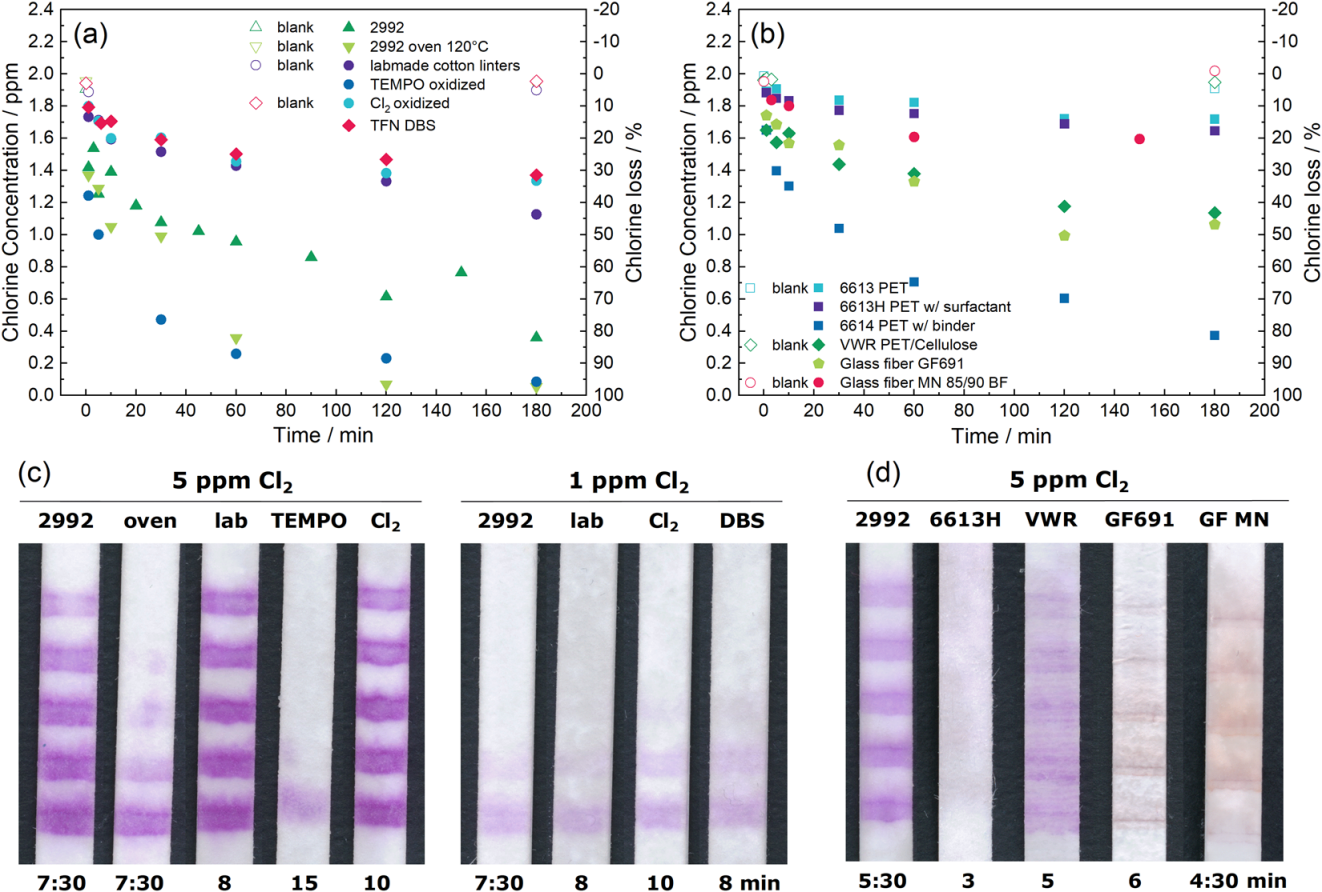
(**a**,**b**) Chlorine loss due to contact with substrate samples over time in a 2 ppm chlorine solution (12 mg substrate per ml solution). The chlorine concentration was determined by the absorbance of the oxidized DPD at 551 nm. “Blank” stands for the concentration of the initial chlorine solution at the beginning and the end of the experiment. (**c**) Images of 9 cm long tests made of buffer-impregnated paper substrates after 5 and 1 ppm free chlorine solution flowed through for the time given below the test. (**d**) Images of 9 cm long tests made of alternative, non-cellulosic substrates without buffer impregnation after 5 ppm free chlorine solution flowed through for the time given below the test. (**a**,**c**) Samples consist of the following paper substrates: Schleicher & Schüll 2992, Schleicher & Schüll 2992 hornified in an oven at 120 °C, lab-made cotton linters paper, TEMPO catalysed pre-oxidized lab-made cotton linters paper, free chlorine pre-oxidized lab-made cotton linters paper (Cl_2_) and DBS paper from Ahlstrom-Munksjö. (**b**,**d**) Samples consist of the following alternative substrates: PET substrates from Ahlstrom-Munksjö 6613 (pure PET), 6613 H (with surfactant) and 6614 (with binder), Spec-Wipe 3 from VWR with 45% polyester and 55% cellulose, glass fibre 691 from VWR and glass fibre MN 85/90 BF from Macherey-Nagel. The flow time is given below each test photo. Please note that the flow time for the Schleicher & Schüll 2992 paper is different for (**c**,**d**) because in (**c**) the paper was used in cross direction (7:30 min) and in (**d**) in machine direction (5:30 min), whereby only the flow time was reduced, and the colour intensity did not decrease significantly. The colour intensity difference here is due to the fact that all tests shown in (**c**) were impregnated with buffer prior printing, all tests in (**d**) not.

Oxidative stability screening was conducted by mixing substrate pieces with a 2 ppm chlorine solution. The remaining chlorine content in the supernatant for different time intervals was analysed photometrically after DPD staining. Figure 5a shows the chlorine loss of solutions after contact with different cellulose substrates. The cotton linters paper 2992 from Schleicher & Schüll, which was used for all the tests presented so far, consumes a substantial amount of chlorine. Already after a contact time of a few minutes, which are needed for the solution to flow through a test, more than 0.5 ppm free chlorine reacted with the paper. Very pure self-made lab cotton linters paper and the DBS paper from Ahlstrom-Munksjö were substantially more stable than the 2992 paper, which indicates that some paper additives in 2992 may be more reactive than the pure cellulose. But as mentioned before, cellulose itself also reacts with chlorine leading to a consumption of 0.5 ppm after 60 min of contact of the chlorine solution with the pure cotton linters papers. Pre-oxidizing cellulose with a concentrated free chlorine solution for 4 h did not increase the stability of the paper as probably only a minor fraction of hydroxyl-groups was oxidized during this treatment. A more effective oxidative treatment using a TEMPO-catalysed oxidation had a substantial effect on the stability of the lab-made paper: Surprisingly, this pre-oxidized paper became more reactive towards chlorine, which could be explained by an increase of surface area exposing more hydroxyl groups for oxidation. To decrease the exposed cellulose surface area, 2992 paper was hornified by placing it overnight at 120 °C in an oven, which should result in irreversible pore closure and adhesion of pore walls to each other^28^. However, also this treatment increased the reactivity of paper further, indicating that the surface processes of cellulose fibres are not yet well understood and need further investigation beyond the scope of this manuscript.

The photometric screening allows to compare the chlorine reactivity of several substrates, whereby the ratio of the chosen 12 mg substrate in contact with 1 ml of 2 ppm chlorine solution is rather random. A 9 cm long and 0.5 cm broad flow test made of 180 g/m² paper, such as the Schleicher & Schüll 2992 paper, weighs 81 mg and wicks roughly 180 µl sample, which would result in a ratio of 450 mg paper for 1 ml of analyte solution flowing through the test. If one considers that only the 0.5 cm end of a test has permanent contact with 0.5 ml chlorine solution, this ratio comes down to 9 mg/ml, which is close to the chosen 12 mg/ml for the screening. Due to this large difference between the two assumptions, the relevance of the substrate screening for the test performance was checked. Figure 5c shows images of flow tests made with the different paper substrates screened in Fig. 5a. Already the analysis of 5 ppm chlorine solution shows large differences between the substrates. While for the more oxidation stable paper substrates all 5 stripes of the barcode test are coloured, the more reactive papers pre-treated by TEMPO-catalysed oxidation or hornification showed much less stripes. Furthermore, the chemical pre-oxidation of the cotton linters led to a substantial increase of the flow time and hence also the contact time of test and analyte solution. A slim intensity difference of the coloured stripes on the 2992 paper and the more stable alternative papers was seen when analysing 1 ppm chlorine. But the differences here were less pronounced than for the less stable substrates. From the test results of the different paper substrates, it becomes clear that the chlorine stability screening is relevant for the test performance and that more inert substrates than cellulose paper are needed for a flow test with very low detection limits.

Figure 5b shows the reactivity of several synthetic bibulous materials when using the same test setup as in Fig. 5a. The most stable material is the PET substrate 6613 by Ahlstrom-Munksjö. While this substrate is not wettable, the version treated with surfactant 6613 H shows bibulous behaviour and is almost as stable. The substrate 6614 contains further a binder which makes this substrate substantially more reactive. Glass fibre substrates showed varying reactivity even though only substrates without binder were used. Figure 5d shows tests after analysis of 5 ppm chlorine with the mentioned substrates. Unfortunately, the dye bleeds substantially on the PET and the GF substrates. The flow on the PET substrate is so fast that almost no purple SA colour remains on this substrate. SA bleeds a little bit less on a substrate containing a mix of PET and cellulose by VWR. However, this material is as reactive as the pure cellulose substrates. SA bleeding is not as strong on the GF substrates than on PET, but the colour intensity is substantially reduced. In summary, it seems that only cellulose paper retains SA in the flow without immobilizing it to the substrate. On the other hand, the PET substrate 6613 H and also the glass fibre from Macherey-Nagel are substantially more inert towards chlorine oxidation and hence desirable to use.

### Improved flow tests with oxidation stable substrates

**Figure 6:**
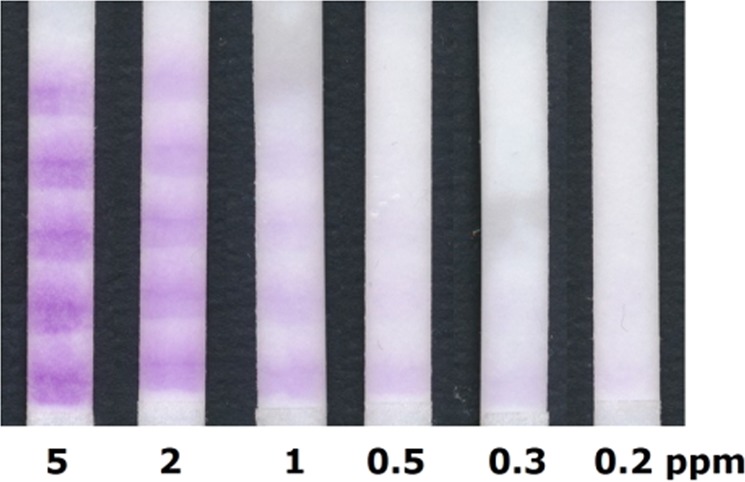
Images of hybrid flow tests with 2992 paper substrate impregnated with pH 5 buffer, 2992 paper wick (4 cm × 0.5 cm) and a sample pad PET 6613 H (1.2 cm × 0.5 cm) after 5 to 0.2 ppm free chlorine solutions flowed through for approximately 5:30 min.

For an optimized performance, a hybrid flow test combines a more oxidation stable substrate (e.g., PET), with a SA retaining cellulose paper (e.g., 2992 paper) such as shown in Fig. 2e. PET is used as a sample pad to avoid chlorine consumption while the analyte is flowing to the first detection line. The SA ink is printed as before on paper to avoid dye bleeding. Indeed, this approach increases the sensitivity compared to tests without an inert sample pad. The test results for different chlorine solutions are shown in Fig. 6, whereby 0.2 ppm of free chlorine can be clearly detected.

**Figure 7:**
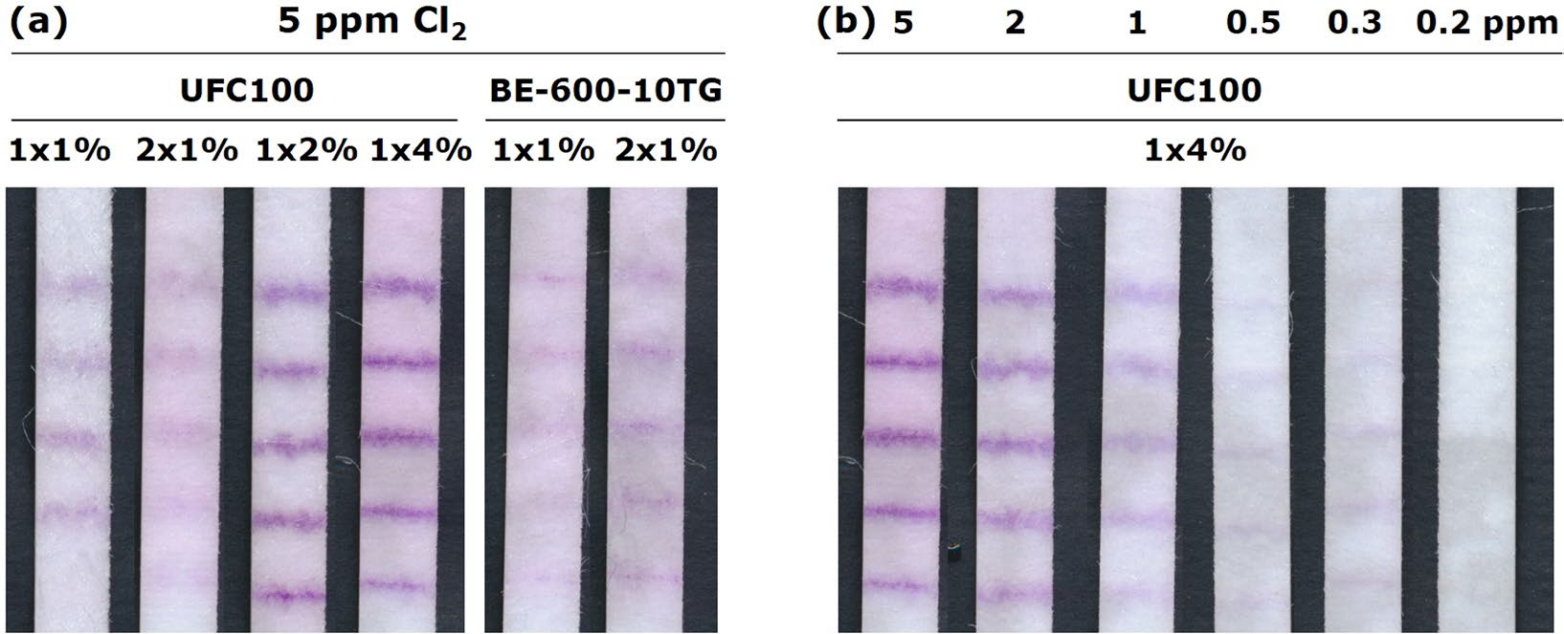
(**a**) Images of flow tests using a PET 6613 H substrate without buffer impregnation and a 2 mM SA ink with 1, 2 or 4% (m/v) cellulose fines UFC100 or BE-600-10TG printed at 2.5 µl/cm for 1 or 2x. A solution with 5 ppm free chlorine flowed through all the tests. (**b**) Images of flow tests with 1 × 4% (m/v) UFC100 in the SA ink after 5 to 0.2 ppm free chlorine solutions flowed through for approximately 2:30 min.

A more pronounced improvement of sensitivity can, however, only be reached if cellulose is substituted by a more inert substrate material. Therefore, a method has to be developed to retain SA on the more inert glass fibre or PET substrates. Since SA has apparently a very high retention on cellulose, a first trial to minimize bleeding on the more inert substrates consisted in the printing of cellulose fines with the SA ink. Figure 7a shows flow tests with PET 6613 H substrates on which a SA ink with different content of either the short fibres BE-600-10TG or the ultrafine cellulose powder UFC100 was printed. After a solution of 5 ppm chlorine run through the tests, clear purple stripes could be observed which was not the case without the cellulose fines (cf. Fig. 5d). Increasing the concentration of the fines in the ink to 2 and 4% improved the line quality, whereby it was not advantageous to increase the amount of cellulose by printing an ink several times (cf. Fig. 7a, 2 × 1%). It is important to note that the cellulose fines are because of their higher surface area more reactive towards chlorine than the paper substrates used before. However, there is no negative effect by increasing the fine concentration as the reaction of chlorine with SA is much faster than with cellulose, and dye and fines are deposited in the same substrate sections. Figure 7b shows images of flow tests on which inks with 4% (m/v) UFC100 were printed. Even though the fines improve the SA retention substantially on PET, 0.2 ppm chlorine cannot be detected. Unfortunately, it is not possible to increase the fines content further without clogging the dispenser needle. Furthermore, especially the larger BE-600-10TG fines settle relatively fast after sonification of the ink, which makes the printing difficult. Hence, the practical limit for UFC100 fines addition was 4% and for BE-600-10TG already 1%. Finally, it was only possible to print inks with fines on the PET but not on the GF substrate as the pores of the latter were too small for a homogenous distribution of the fines within the substrate.

The encapsulation with polymers^8,11,12,29^ is a common method to immobilize reagents in a flow test. Hence, we added a variety of different typical polymers such as polyvinylpyrrolidone (PVP), polyethylene glycol (PEG), polyvinyl alcohol (PVA) and also cellulose derivatives such as carboxy methyl cellulose (CMC) and cellulose acetate (CA) to the SA ink instead of cellulose fines. All inks were tested on paper, GF and PET substrates. On paper, there was either no or even a negative effect on the sensitivity due to the polymers. Since the cellulose substrate already prevents SA from bleeding, the polymers can only hinder the coloration of the dye. PVP, PVA and CA reduced the bleeding of the dye on GF. Interestingly, CA also improved the SA colour from orange to more purple similar to the colour on the paper substrates (cf. Fig. 5d). CA was also the only polymer tested, which improved the dye retention substantially on PET and was hence selected for further studies.

**Figure 8:**
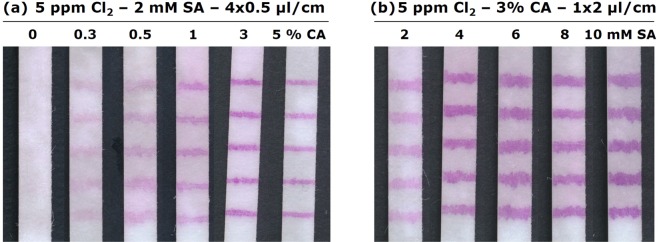
(**a**) Images of flow tests using a PET 6613 H substrate and a 2 mM SA ink with cellulose acetate content of 0 to 5% (m/v) printed 4x at 0.5 µl/cm. (**b**) Images of flow tests using a PET 6613 H substrate and a SA ink of 2 to 10 mM with 3% (m/v) cellulose acetate content printed 1x at 2 µl/cm. A solution with 5 ppm free chlorine flowed through all the tests.

First, the optimum CA concentration in the SA ink was determined. Figure 8a shows PET flow tests after analysing 5 ppm chlorine solutions. As expected, an increasing CA concentration reduces the bleeding. However, the ink gets more and more viscous with increasing CA content and the coloration of the dye on the test slows down as the CA layer must be penetrated first. A content of 3% (m/v) CA in the ink was both for the PET and the GF substrate the most ideal. Since CA cannot be dissolved in ethanol, we used acetone as solvent. Acetone has unfortunately a very high vapor pressure. The evaporation of acetone during the printing process led sometimes to clogging of the syringe needle. Printing directly 2 µl/cm on the substrates instead of 4 × 0.5 µl/cm reduced this problem due to the shorter printing time. However, this change led to an increase of the line width (cf. Fig. 8b) and hence a colour intensity reduction. Fortunately, SA solubility in acetone is not limited to a little more than 2 mM such as in ethanol. Increasing the SA concentration to 4 mM resulted in lines with the same colour intensity than a 2 mM ink printed 4 × 0.5 µl/cm. A further increase in SA concentration did not lead to a further intensity increase. Hence, we continued to print 2 µl/cm of an ink with 4 mM SA and 3% (m/v) CA in acetone. Such tests allowed us for the first time to see a purple hue at a chlorine concentration of 0.1 ppm.

**Figure 9:**
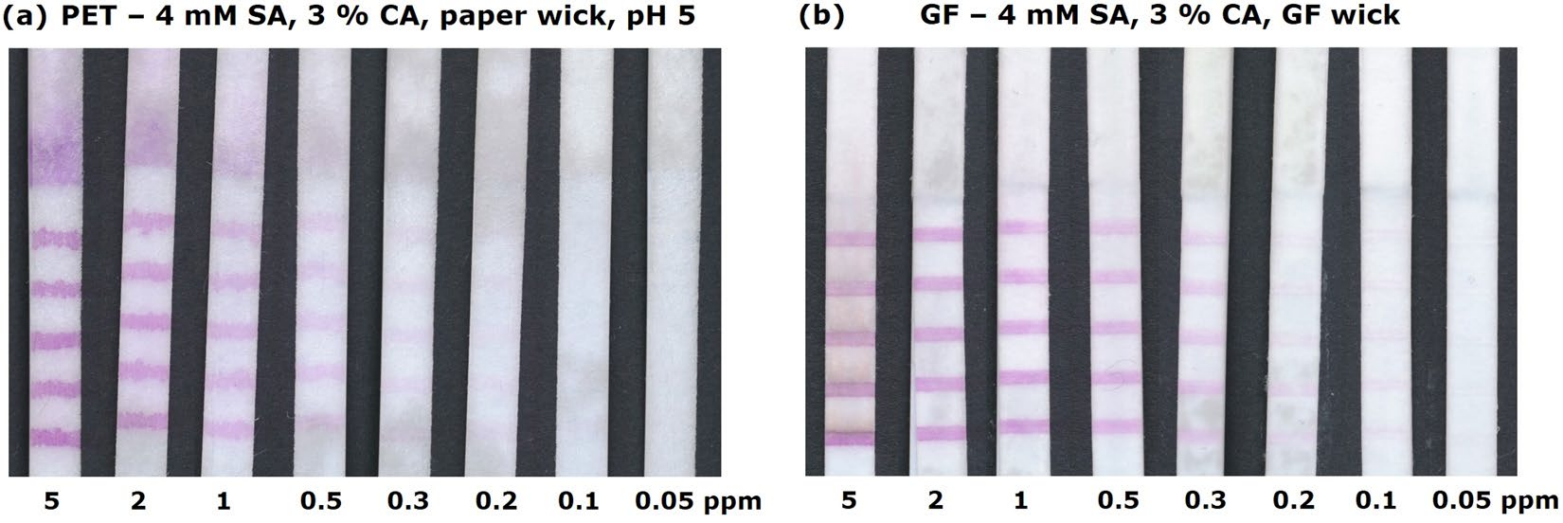
Images of flow tests after 5 to 0.05 ppm free chlorine solutions flowed through for 4–5 min. (**a**) Flow tests with PET 6613 H substrate impregnated with buffer pH 5 and including a 4.5 cm long paper wick. (**b**) Flow tests with GF substrate (MN 85/90 BF) without buffer impregnation but with a 4.5 cm long additional GF wick. The ink consisted for all tests of 4 mM SA with 3% (m/v) CA in acetone, which was printed 1x at 2 µl/cm.

As discussed above, the addition of a buffer and wick increases the sensitivity of the test on paper (cf. Fig. 4). In order to check whether these measures also increase the sensitivity on PET and GF, flow tests with buffer impregnation and/or paper, GF or PET wick were tested. While the impregnation with buffer increases the colour intensity on PET based tests, no positive effect was found for GF tests. The use of an additional wick was beneficial for both substrate materials. For PET, the paper wick led to a higher sensitivity than the PET wick which can probably be explained by the larger sample volume and slower flow time of the paper wick. The GF wick was, however, more effective for the GF substrate, even though it has roughly the same pore volume and a higher flow speed than the paper wick. Images of these optimized flow tests on GF and PET after different solutions with 5 to 0.05 ppm chlorine content flew through are shown in Fig. 9. A slight purple hue is visible for 0.1 ppm chlorine with the PET tests, and even for 0.05 ppm with the GF tests. It is worth to note that all SA lines are coloured simultaneously whereby the number of lines depended on the chlorine concentration for the paper-based tests (cf. Figs 3 and 6). Hence, it seems that the coloration of the stripes on the paper depended more on the consumption of chlorine during the flow by the substrate than a depletion of chlorine in the analyte solution due to reaction with SA. Interestingly, a broad 6^th^ line appears for high chlorine concentrations on the PET substrate (Fig. 9a). This purple pad is due to the paper wick which catches bleeding SA and appears as a 6^th^ line because the intensity is strong enough to shine through the PET substrate. This effect can either be used for a distinction between different high chlorine concentrations, but also removed from the user by sheathing the flow test strip. Since for the stable substrates all lines are coloured simultaneously, the sensitivity is the same for tests with only one line instead of five. However, printing several lines is still beneficial as in case of very low concentrations the coloration of several lines can be evaluated to confirm the visual impression of the first.

## Conclusion

**Figure 10:**
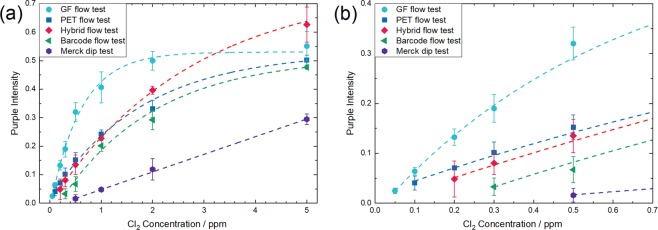
(**a**) Intensity of the purple SA dye in dependence of the free chlorine concentration extracted from the different chlorine tests presented in the previous figures: GF flow test (Fig. 9b), PET flow test (Fig. 9a), Hybrid flow test (Fig. 6), Bar code flow test (Fig. 3) and Merck dip test (Fig. 3). To determine the error of the analysis, the purple intensity was extracted of images of three independently run flow tests for each concentration of which the average and the standard deviation was determined. The dashed lines were integrated for guidance of the eye and were obtained by an asymptotic exponential fit (y = a − bc^x^) of the data sets. (**b**) Zoom of (**a**) in the range of 0–0.7 ppm free chlorine to see the differences for low concentrations.

A summary of the performance of the different test designs discussed in this article is shown in Fig. 10, which displays the intensity of the first purple line of the five different main test types after analysis of solutions between 5 and 0.05 ppm free chlorine. The commercial dip test available from Merck KGaA is currently sold with a detection range between 0.5 and 20 ppm free chlorine. Interestingly, the colour intensity depends linearly from the chlorine concentration in the tested range. As all the newly developed flow tests are substantially more sensitive, their linear detection range ends already at 1 ppm or even at 0.5 ppm chlorine for the most sensitive GF-based test. On the other hand, the GF test detects as low as 0.05 ppm chlorine, which is 10x lower than the lower end of the detection range of the commercial dip test. From the results of our studies it can be concluded that a substantial improvement originates already from the change from a dip to a flow test format. The impregnation of a paper substrate with a pH 5 buffer leads furthermore to a more intense purple line colour for high concentrations but has little impact for low concentrations. Adding an additional wick and an inert sample pad to a flow test to build a hybrid flow test shifts the detection limit even further to smaller chlorine concentrations. In order to detect very low free chlorine concentrations, an oxidation stable substrate material has to be used. Tests with GF substrates have a higher colour intensity than with PET substrates. That’s why GF tests show also a colour intensity saturation at lower chlorine concentrations. The developed GF and PET based flow tests with CA to avoid SA bleeding allow to determine as low as 0.05 or 0.1 ppm free chlorine within 4–5 min flow time, whereby no user action is needed during the analysis. It is evident that the latter exceeds by far the possible detection limit of commercial dip tests and therefore are promising candidates for advanced applications where a detection of trace amounts of free chlorine is crucial.

## Data Availability

The datasets supporting the findings of this study are available from the corresponding author on reasonable request.
